# High-Productivity Continuous Conversion of Glucose to α-Hydroxy Esters over Postsynthetic and Hydrothermal Sn-Beta Catalysts

**DOI:** 10.1021/acssuschemeng.1c06989

**Published:** 2022-03-30

**Authors:** Luca Botti, Ricardo Navar, Søren Tolborg, Juan S. Martínez-Espín, Ceri Hammond

**Affiliations:** † Department of Chemical Engineering, Imperial College London, London SW7 2AZ, U.K.; ‡ Cardiff Catalysis Institute, Cardiff University, Park Place, Cardiff CF10 3AT, U.K.; § Biobased Chemicals R&D, Haldor Topsøe A/S, Haldor Topsøes Allé 1, 2800 Kgs. Lyngby, Denmark

**Keywords:** biomass, zeolite, continuous, retro-aldol, Sn-Beta

## Abstract

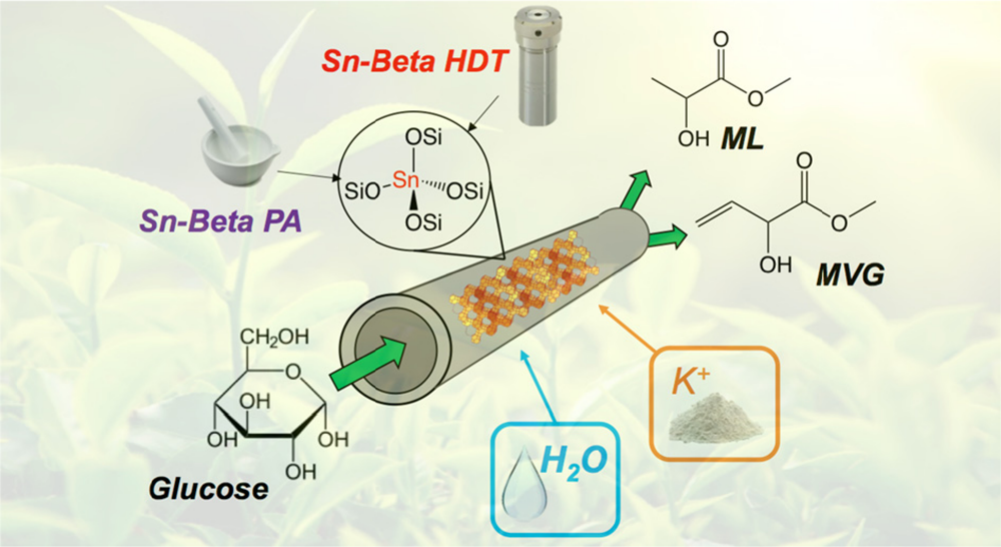

The retro-aldol fragmentation of glucose is a complex reaction of industrial relevance, which provides a potentially sustainable route for the production of α-hydroxyester compounds of relevance to the green polymer industry, such as methyl lactate and methyl vinyl glycolate. Although the zeolite catalyst, Sn-Beta, has shown itself to be a promising catalyst for this process, important information concerning the stability of the catalyst during continuous operation is not yet known, and improvements to its yield of retro-aldol products are also essential. Here, we perform detailed spectroscopic studies of a selection of Sn-Beta catalysts and evaluate their performances for the retro-aldol fragmentation of glucose under continuous processing conditions, with the dual aims of developing new structure–activity–lifetime relationships for the reaction and maximizing the productivity and selectivity of the process. Kinetic studies are performed under both established reaction conditions and in the presence of additional promoters, including water and alkali salts. Generally, this study demonstrates that the reaction conditions and choice of catalyst cannot be optimized in isolation, since each catalyst explored in this study responds differently to each particular process perturbation. However, by evaluating each type of the Sn-Beta catalyst under each set of reaction conditions, we reveal that postsynthetic Sn-Beta catalysts exhibit the best levels of performance when activity, selectivity, and stability are taken into account. Specifically, the best levels of performance are obtained with a postsynthetic Sn-Beta catalyst that is preactivated in a flow of methanol prior to reaction, which provides α-hydroxyester yields over 90% at the early stages of continuous operation and operates at high yield and selectivity for over 60 h on stream. Space–time-yields over two orders of magnitude higher than any previously reported for this reaction are achieved, setting a new benchmark in terms of the retro-aldol fragmentation of glucose.

## Abstract 2

Continuous conversion of renewable glucose to industrially relevant alpha-hydroxy esters is achieved by use of Sn-containing zeolite catalysts.

## Introduction

Climate change and the depletion of nonrenewable resources are increasing the need to replace common fossil feedstock with more sustainable alternatives.^1,2^ In this light, the chemical industry is increasingly directing its attention toward biomass as a renewable source of carbon for the production of chemical commodities.^3−5^ Although several approaches are undergoing intense study, a major challenge is to develop methods to catalytically convert abundant monosaccharide sugars into useful chemicals. Given that glucose is the cheapest and most abundant monosaccharide sugar that can be gathered from agricultural waste biomass, its use as a feedstock is of particular interest.^6,7^

One of the most interesting catalysts for glucose upgrading is the Lewis acidic zeolite, Sn-Beta.^8−12^ At a relatively low temperature (90–110 °C), Sn-Beta is known to catalyze the isomerization of glucose to fructose, which is an important reaction in the food industry and for future biorefineries.^9,13,14^ At higher temperatures (150 °C and above), Sn-Beta is known to promote the retro-aldol fragmentation of mono- and di-saccharides to α-hydroxy ester compounds, including methyl lactate (ML) and methyl vinyl glycolate (MVG).^15,16^ These α-hydroxy ester compounds have attracted significant industrial interest due to their potential as monomers and precursors for several surfactants.^17−19^

However, several aspects of the retro-aldol fragmentation of monosaccharides are yet to be fully understood or optimized. For example, in previous studies, the monosaccharide fructose has primarily been used as the substrate, due to its higher solubility and reactivity compared to glucose.^20^ Hence, these reports do not directly address the retro-aldol conversion of glucose, which is the most abundant and cheapest monosaccharide available for future biorefining.^16,21^ Furthermore, although studies have shown how the selectivity of Sn-Beta for retro-aldol product formation can be enhanced by addition of alkali salts to the system, combined yields of ML and MVG have rarely exceeded 60% when monosaccharides are used as substrates, resulting in an undesirable loss of valuable feedstock.^12,18−22^ Finally, previous studies focusing on the retro-aldol conversion of glucose have been performed at dilute catalyst concentrations in batch reactors, which is not conducive for achieving high levels of reactor productivity and does not provide essential information on the stability of the catalyst, which is one of the most important catalyst performance indicators.^23−26^ In regard to this latter point, although a small number of studies have recently started to address the continuous performance of Sn-Beta for retro-aldol product formation, several specific hurdles remain. For example, although Zhang et al.^27^ recently addressed the continuous conversion of glucose to ML in a continuous flow reactor, their study was performed at very low weight hourly space velocities (eq 1, WHSV, 0.1224 h^–1^), which results in low volumetric productivities being achieved and could mask intrinsic deactivation of the catalyst by operating in an “excess catalyst” regime.^24,25^ Likewise, although we recently demonstrated how the addition of small amounts of water to the feed could increase the productivity and stability of Sn-Beta for retro-aldol product formation during continuous operation, the beneficial role of water was only demonstrated when using fructose as the substrate at a WHSV of 0.45 h^–1^.^16,26^ As such, the intrinsic stability of Sn-Beta for the continuous retro-aldol fragmentation of glucose to α-hydroxyesters is not yet known, and high-productivity performance has not yet been established.
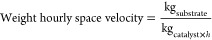
1

In addition to developments from the process side, further developments are also necessary for the catalyst preparation side. Generally, the hydrothermal synthesis of Sn-Beta is known to generate an active and selective catalyst for retro-aldol fragmentation, due to the particular active site speciation and the defect-free nature of its hydrophobic framework.^28−30^ However, despite its typically excellent levels of performance, the synthesis of this material has several drawbacks that complicate its industrial use.^31−33^ Therefore, many studies have focused on the synthesis of Sn-Beta materials made by alternative, postsynthetic procedures.^8,32−38^ In these, a commercially available parent aluminosilicate zeolite is converted into Sn-Beta by removal of the original aluminum atoms, followed by reinsertion of Sn in a second synthesis step. Although several breakthroughs have been made in this regard, postsynthetic Sn-Beta materials have rarely exceeded the performance of hydrothermal Sn-Beta.^28,29^ Consequently, the synthesis of better performing postsynthetic Sn-Beta materials and evaluation of their performances for retro-aldol fragmentation under continuous processing conditions are important targets.

Recently, we reported how the performance of postsynthetic Sn-Beta materials prepared by solid-state incorporation (SSI) could be improved by “preactivation” prior to reaction.^39^ Preactivation involved treatment of the synthesized catalyst in a flow of methanol at 110 °C for 0.5 h, followed by calcination in air prior to operation. This procedure resulted in substantial improvements to the activity and stability of Sn-Beta for the isomerization of glucose to fructose.^38^ In particular, the activity of the catalyst was found to increase by a factor of two following preactivation, and its stability was also found to be enhanced by this procedure, making this catalyst a promising postsynthetic alternative to hydrothermal Sn-Beta.

Herein, we fully interrogate the catalytic performance of preactivated Sn-Beta and compare its properties and performance to both its postsynthetic parent material, i.e., Sn-Beta prepared by classical SSI, and also to hydrothermally prepared Sn-Beta, for glucose isomerization and the retro-aldol fragmentation of glucose to ML and MVG. The properties of each catalyst are investigated by means of ^119^Sn MAS NMR, diffuse reflectance infrared Fourier transform spectroscopy (DRIFTS), vapor sorption, and porosimetry. The performance of each catalyst is evaluated in the absence and presence of different activity promoters, such as alkali and water, to fully evaluate the performance of each material, while aiding the development of structure–activity correlations.^12,16,21,22^ To better represent industrial operational conditions while also providing important details regarding the stability of each catalyst, kinetic testing is primarily performed in the continuous regime in plug flow reactors (PFRs).^23−25,39−41^ In addition to facilitating the understanding of the properties and performance of preactivated Sn-Beta, the second aim of this research is to improve the productivity, selectivity, and stability of the retro-aldol process starting from glucose, as opposed to more reactive monosaccharides, such as fructose. Through this research, we achieve space–time-yields that are over two orders of magnitude higher than previously reported in the open literature, providing a new benchmark in terms of Sn-Beta-catalyzed retro-aldol fragmentation reactions.

## Results and Discussion

### Catalyst Synthesis and Characterization

**Scheme 1:**
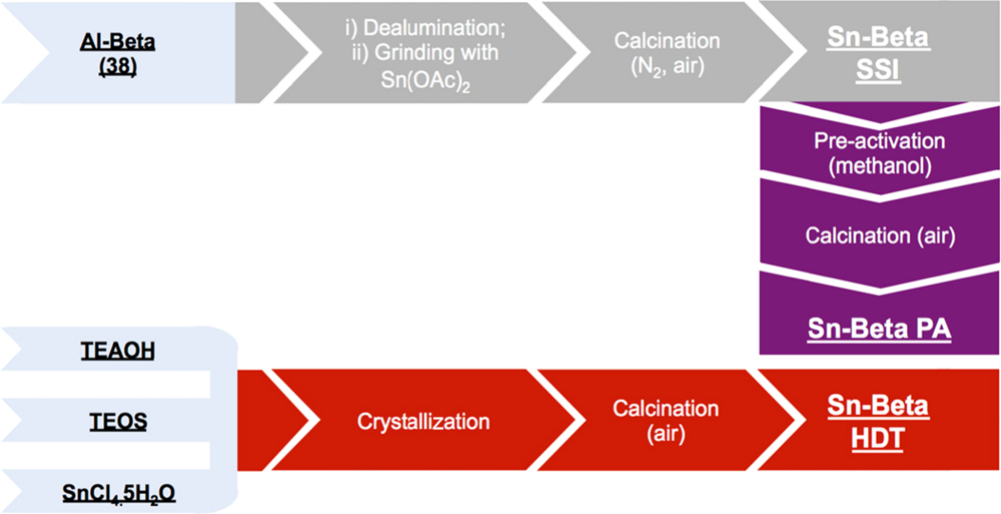
Overview of the Synthesis Routes Explored in the Manuscript for the Preparation of Sn-Beta

Three Sn-Beta samples were prepared by various synthetic procedures. First, a sample of Sn-Beta was prepared by classical hydrothermal synthesis (henceforth denoted as Sn-Beta HDT), using fluoride as the mineralizing agent and tetraethylammonium hydroxide as the organic structure directing agent.^41^ Alongside this, a sample of postsynthetic Sn-Beta was prepared by the SSI of tin(II) acetate into the vacant lattice sites of dealuminated zeolite Beta (original Si/Al = 19, dealuminated in hot HNO_3_ solution, see the Experimental Details Section for details).^28,32^ Half of the postsynthetic sample was subsequently preactivated according to our recently identified procedure, which involves treatment in a flow of methanol for 0.5 h at 110 °C, followed by calcination in air at 550 °C for 3 h.^38^ These samples were denoted as Sn-Beta SSI and Sn-Beta PA, respectively, as summarized in Scheme 1. All samples were prepared with a final Sn loading of 1 wt % Sn, corresponding to an Si/Sn molar ratio of 200 in the catalyst (Table 1).

**Table 1:** Textural Properties of Sn-Beta HDT, PA, and SSI

catalyst	BET area (m^2^ g^–1^)^a^	total pore volume (cm^3^ g^–1^)^b^	Si/Sn molar ratio^c^
Sn-Beta HDT	398	0.21	202
Sn-Beta SSI	581	0.24	206
Sn-Beta PA	564	0.23	198

a Surface area determined from nitrogen adsorption using the BET equation.

b Micropore volume determined by the t-plot method.

c Determined by ICP-MS measurements. All other experimental details are provided in the Experimental Details Section.

Textural analysis of each synthesized catalyst (Sn-Beta SSI, Sn-Beta PA, and Sn-Beta HDT) by XRD and porosimetry showed that each catalyst possessed a crystalline, microporous structure, characteristic of the BEA framework, confirming the successful synthesis of each material (Table 1 and Figure S1). The main textural differences among these materials related to the dimensions of the crystallites.^28^ In particular, it has previously been demonstrated that Sn-Beta HDT exhibits crystallite sizes of the order of 10–15 μm, whereas postsynthetic Sn-Beta samples typically possess crystal sizes of 0.5–2 μm, in line with previous reports that have demonstrated that zeolite synthesis in fluoride media results in the formation of larger crystallites.^28^ The larger crystallites of Sn-Beta HDT likely account for the slightly lower surface area and micropore volume of Sn-Beta HDT compared to Sn-Beta SSI and Sn-Beta PA (Table 1).

To compare the active site distributions of the three catalyst materials, ^119^Sn MAS NMR spectroscopy analysis was performed. In line with our recent studies, analyses were performed with a Carr–Purcell–Meiboom–Gill (CPMG) echo train acquisition method, adapted from the protocol demonstrated for Sn-Beta samples by the Ivanova group.^43,44,56^ Under these conditions, the technique has been shown to be able to effectively distinguish different species of Sn by their chemical environment and coordination. The technique was performed on hydrated materials to emulate the state of the catalyst upon placement into the reactor, and a long recycle delay (d1 = 135 s) was employed, to provide a quantitative overview of the different Sn species present in each material.^39^

**Figure 1:**
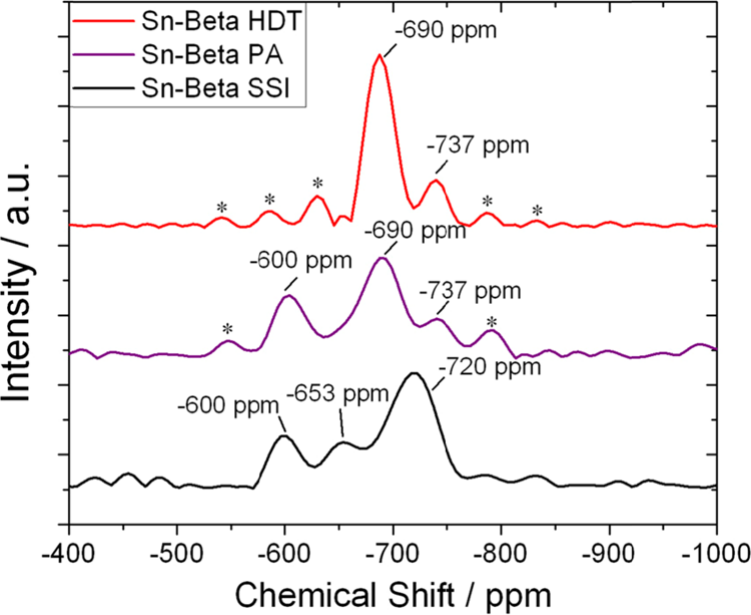
^119^Sn CPMG MAS NMR spectrum of various Sn-Beta samples prepared by hydrothermal synthesis (Sn-Beta HDT, top line) and postsynthetic methods (Sn-Beta PA, middle line and Sn-Beta SSI, bottom line). (Recycle delay d1: 135 s). Spinning side bands are marked with an asterisk.

Figure 1 presents the ^119^Sn CPMG MAS NMR spectra of Sn-Beta HDT (top line), Sn-Beta PA (middle line), and Sn-Beta SSI (bottom line). The resonance at −600 ppm is typical of extra-framework Sn species, typically tin oxide, whereas signals at lower chemical shift values are known to be related to Sn species present within the zeolite lattice but present in a hydrated form.^45^ In line with previous reports, Sn-Beta HDT did not present a detectable resonance at −600 ppm, indicating that extra-framework Sn in this material was either absent or was present below the detectability limit of the measurement.^46^ In contrast, some extra-framework Sn was observed for both postsynthetic samples, accounting for approximately 25–30% of the total signal. The absence of extra-framework Sn in Sn-Beta HDT can be attributed to a more effective incorporation of tin by the hydrothermal methodology, in line with previous reports.^28,43^

Although the dominant resonance in all samples was found in the region attributable to isomorphously substituted Sn species (−650 to −750 ppm), differences in the chemical shift and intensity of the signals were observed. In Sn-Beta HDT, a main resonance at −690 ppm was observed, alongside a less intense resonance at −737 ppm. Sn-Beta SSI, on the other hand, exhibited a main peak at −720 ppm and a second resonance at −650 ppm. However, preactivation of the postsynthetic catalyst caused the −650 ppm resonance to be lost, the dominant resonance to shift from −720 to −690 ppm, and a new resonance at −737 ppm to be formed. However, the SnO_2_ content present in the postsynthetic material was not impacted by preactivation, resulting in Sn-Beta PA containing a similar quantity of SnO_2_ to Sn-Beta SSI.

The molecular understanding of how the difference in chemical shift relates to the properties of hydrated Sn species is the topic of debate in the scientific community. Although diverse and sometimes contradictory assignments are reported in the literature, it is reasonable to assume that the shifting of these signals are related to changes in the active site properties such as T-site location, Sn–O–Si bond angle, and/or degree of hydration.^46,47^ In this light, it is notable how preactivation of Sn-Beta SSI modifies the active centers of the postsynthetic catalysts in such a way that they exhibit NMR signals comparable to those found in the hydrothermal sample. This finding suggests that common active site properties may be found for these catalysts, despite being prepared by very different preparation procedures.

**Figure 2:**
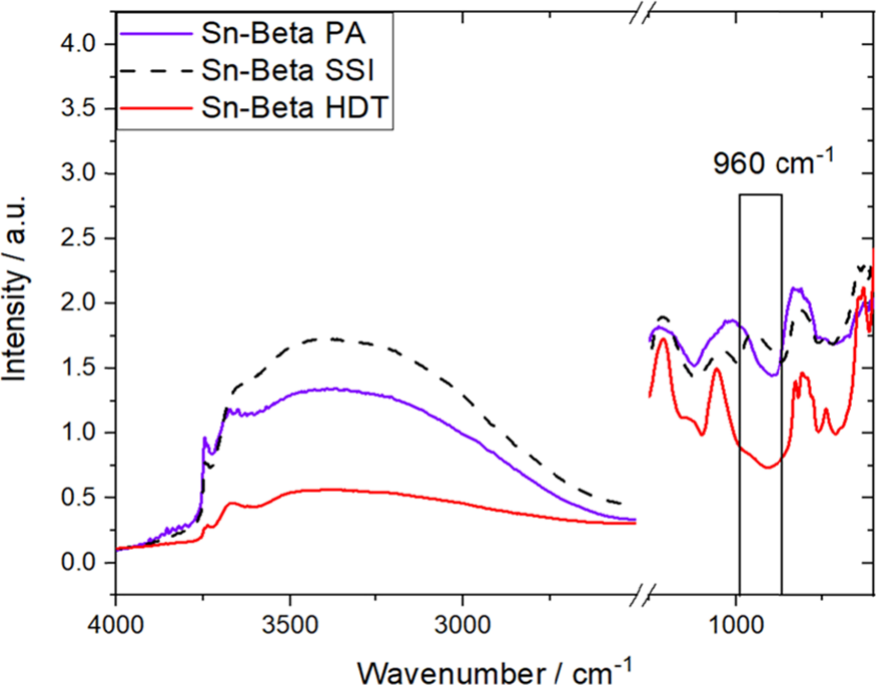
DRIFT spectra of Sn-Beta SSI (dashed line), Sn-Beta PA (middle, violet line), and Sn-Beta HDT (bottom, red line) recorded at 25 °C.

To determine the lattice properties of each catalyst and hence to identify how preactivation affected the properties of the zeolite framework, study of the three catalysts was also undertaken by DRIFTS (Figure 2). As can be seen, the spectrum of Sn-Beta PA shows an increased contribution of isolated silanol groups (≡Si–OH, 3740 cm^–1^), a decrease in the magnitude of H-bonding interactions (3200–3600 cm^–1^),^48,49^ and a decrease in intensity characteristic of internal defect sites (960 cm^–1^), when compared to the spectrum of Sn-Beta SSI.^50^ Taken together, these changes indicate that the postsynthetic material becomes less defective and more hydrophobic following preactivation. Interestingly, Sn-Beta HDT already exhibited very low signals in these regions, in agreement with previous reports that have shown that this material possesses a low number of defect sites and larger crystallite sizes.^28^

**Figure 3:**
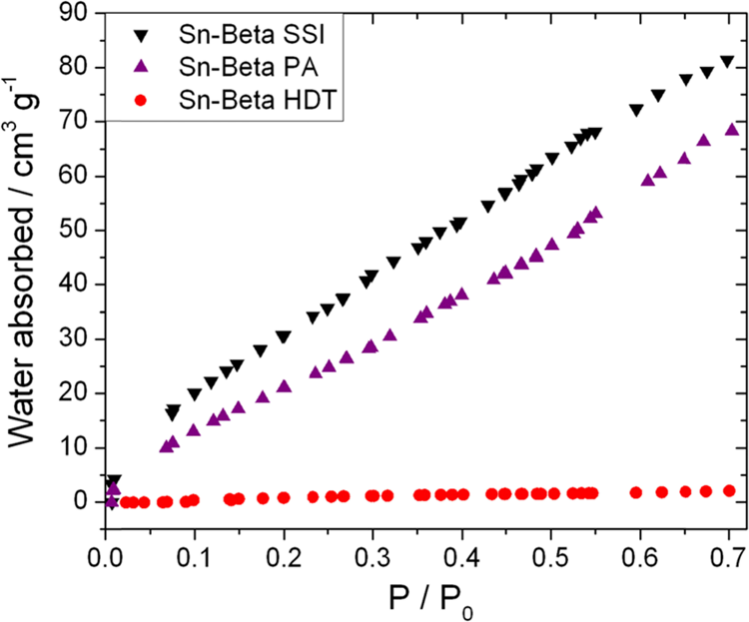
Water sorption isotherms of Sn-Beta SSI (top, downward triangles), Sn-Beta PA (middle, upward triangles), and Sn-Beta HDT (bottom, circles). Isotherms gathered at 20 °C from 0 to 0.7 *P*/*P*_0_.

To further evaluate the differences in hydrophilic properties between the samples, vapor sorption studies were performed using water as the adsorbate (Figure 3). This analysis revealed drastic differences between Sn-Beta HDT and the postsynthetic Sn-Beta samples. In particular, Sn-Beta HDT adsorbed up to 20 times less water than postsynthetic Sn-Beta materials, confirming the highly hydrophobic nature of this material. Although both postsynthetic samples adsorb much higher quantities of water, it is notable that Sn-Beta PA demonstrated a substantial (ca. 30%) decrease in hydrophilicity in comparison to Sn-Beta SSI. Thus, Sn-Beta PA possesses a different active site structure from Sn-Beta SSI and is also more hydrophobic than the parental Sn-Beta SSI material. Taken together, catalyst characterization suggests that preactivation modifies the active site and the textural properties of postsynthetic Sn-Beta. In particular, the resulting sample (Sn-Beta PA) possesses active Sn sites more comparable to Sn-Beta HDT, albeit with some residual extra-framework Sn present from the original synthesis, but it has textural properties (cf. hydrophilicity and defect sites) more comparable to those of Sn-Beta SSI but with enhanced levels of hydrophobicity.

### Glucose Isomerization

**Figure 4:**
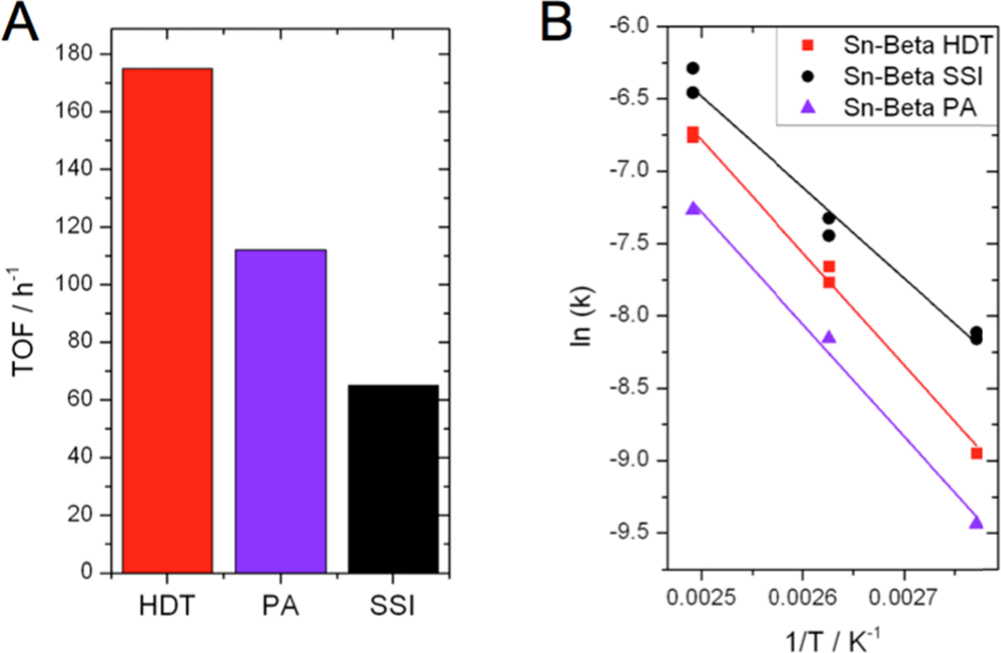
(A) TOF value for continuous glucose isomerization performed in pure methanol for Sn-Beta HDT, Sn-Beta PA, and Sn-Beta SSI, calculated at 0.5 h of time on stream. Reaction conditions: 1 wt % glucose in methanol, flow rates of 1.5 mL min^–1^ (Sn-Beta HDT), 1.0 mL min^–1^ (Sn-Beta PA), and 0.6 mL min^–1^ (Sn-Beta SSI), 100 mg of catalyst, 110 °C, 15 bar. (B) Arrhenius expression obtained for glucose isomerization for Sn-Beta HDT (red squares), Sn-Beta SSI (black circles), and Sn-Beta PA (violet triangles). Reaction conditions: 1 wt % glucose in methanol, 10 min reaction time, 4 mL reaction volume, mol glucose/mol Sn of 50, reaction temperatures of 90, 105, and 120 °C.

The activity of Sn-Beta for glucose isomerization (GI) is dependent on its water affinity and active site speciation.^28,29,39,51^ Therefore, due to the differing hydrophilicity and active site environment(s) of the three Sn-Beta materials described above, preliminary analysis of their kinetic performance was undertaken by use of glucose isomerization. In particular, the performances of Sn-Beta PA, Sn-Beta HDT, and Sn-Beta SSI were evaluated for continuous GI at 110 °C using a reaction feed of 1 wt % glucose in pure methanol, in line with previous studies (Figure 4).^28,39^ The initial activity of each catalyst was evaluated considering the percentage of glucose converted at 0.5 h of time on stream and was normalized to account for the different WHSVs of each reaction by transformation to a turnover frequency (TOF, eq 2).^28,39^ Among the three catalytic materials, Sn-Beta HDT exhibited the highest initial activity, achieving a TOF of 175 h^–1^. Although both postsynthetic samples were lower in activity than Sn-Beta HDT, Sn-Beta PA (112 h^–1^) exhibited twice the activity of Sn-Beta SSI (64 h^–1^), demonstrating the extent of improvement in activity that occurs upon preactivation of the SSI catalyst in a flow of methanol.
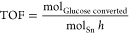
2

In general, the best catalyst for GI possesses the lowest affinity for water (Figure 3). However, the correlation between vapor sorption and activity is not strong, given that Sn-Beta PA exhibited twice the activity of Sn-Beta SSI yet was only approximately 30% more hydrophobic. Furthermore, despite Sn-Beta HDT being approximately 20 times more hydrophobic than Sn-Beta PA, its activity was only around 30% higher than that of Sn-Beta PA. However, on a broader level, the general observation is in agreement with recent findings, which revealed how more hydrophobic Sn-Beta catalysts ensure preferential interaction of the active site with the substrate.^29,52^ This preferential interaction is reportedly achieved by the fact that more hydrophobic materials strongly hinder access of the polar solvent molecules (such as alcohols or water) to the active site, thus avoiding competition between the solvent and the substrate for coordination to the Sn site. Interestingly, Gounder et al.^29^ reported how this increment in activity due to exclusion of the polar solvent was accompanied by an increase in the activation energy. The authors hypothesized that this increase in activation energy occurred since stabilization of the transition state by solvent molecules in the vicinity of the active site was lost upon exclusion of the solvent for hydrophobic materials. Yet, despite this loss of transition state stabilization, increased absorption on the substrate onto the Sn active site due to the absence of the reaction solvent resulted overall in increased activity for Sn-Beta materials that exhibited higher levels of hydrophobicity.

To further probe this hypothesis and determine a potential explanation for the improved activity of Sn-Beta PA versus Sn-Beta SSI, the activation energies and the pre-exponential factors for Sn-Beta HDT, Sn-Beta PA, and Sn-Beta SSI were determined for GI between 90 and 120 °C (Figures 4B and S2). These specific kinetic experiments were performed in batch reactors, so that intrinsic kinetic data could be directly determined without contribution from catalyst deactivation or limitations that can be found by working at high levels of reactant conversion.^23^ All Arrhenius parameters were determined according to the quantity of glucose converted. As can be seen (Table 2), Sn-Beta HDT has an activation energy 18 kJ mol^–1^ higher than that of Sn-Beta SSI and a pre-exponential factor (A) two orders of magnitude larger.^52^ The higher activation energy suggests a less facile mechanism for isomerization over Sn-Beta HDT than for Sn-Beta SSI, but the accompanying increase in the pre-exponential factor indicates that the entropic contributions to reaction are more favorable for the hydrothermal catalyst, such as Sn-site location, substrate accessibility, and reactivity.^53^ These observations are in good agreement with the recent studies of Gounder and coworkers.^29^

**Table 2:** Values for Activation Energy (*E*_a_) and Pre-Exponential Factor (A) Extrapolated from the Arrhenius Expression Shown in Figure 5 for Sn-Beta HDT, Sn-Beta SSI, and Sn-Beta PA^a^

catalyst	*E*_a_ (kJ mol^–1^)	A (s^–1^)
Sn-Beta HDT	86 ± 3	(3.5 ± 2) × 10^8^
Sn-Beta PA	86 ± 6	(2 ± 4) × 10^8^
Sn-Beta SSI	70 ± 6	(3 ± 4) × 10^6^

a Reaction conditions: 1 wt % glucose in methanol, 10 min reaction time, 4 mL reaction volume, mol glucose/mol Sn of 50, reaction temperatures of 90, 105, and 120°C.

Interestingly, preactivation of Sn-Beta clearly increased the activation energy of the postsynthetic catalyst, from 70 kJ mol^–1^ for Sn-Beta SSI to 86 kJ mol^–1^ for Sn-Beta PA. Furthermore, preactivation also increased the pre-exponential factor by two orders of magnitude (Table 2). Thus, preactivation not only results in a twofold increase in catalytic performance but also results in the Arrhenius parameters of Sn-Beta PA matching those of Sn-Beta HDT. This observation strongly indicates that Sn-Beta PA and Sn-Beta HDT possess common active site properties, which are characterized by increased activation energies relative to Sn-Beta SSI, but improved entropic characteristics. This finding is in excellent agreement with the findings from ^119^Sn MAS NMR spectroscopy, which indicated that the preactivated sample possessed active Sn sites much more comparable to those of Sn-Beta HDT than its postsynthetic parent material, Sn-Beta SSI. The increment in activity and temperature dependence after preactivation, alongside a lower affinity for water absorption, allow us to hypothesize that preactivation results in the formation of an improved material that experiences better interaction between the substrate and active site of reaction, due to lower levels of competitive absorption with the reaction solvent, unlocking in this way improved overall performances. However, although preactivation leads to a twofold increment in activity for GI after preactivation (Figure 4), it is notable that Sn-Beta PA still exhibits somewhat lower activity in comparison to Sn-Beta HDT for GI, when evaluated on a TOF basis.^28,39^ We hypothesize that this is likely due to the extra-framework SnO_2_ content of this material, which is present from the original SSI synthesis and does not appear to change in abundance during preactivation (Figure 1). The presence of residual extra-framework SnO_2_ would evidently decrease the apparent TOF of this catalyst relative to the hydrothermal sample, given that such species are known to be spectators in the context of Lewis acid catalysis.^54^

### Glucose Upgrading to α-Hydroxy Esters

**Scheme 2:**
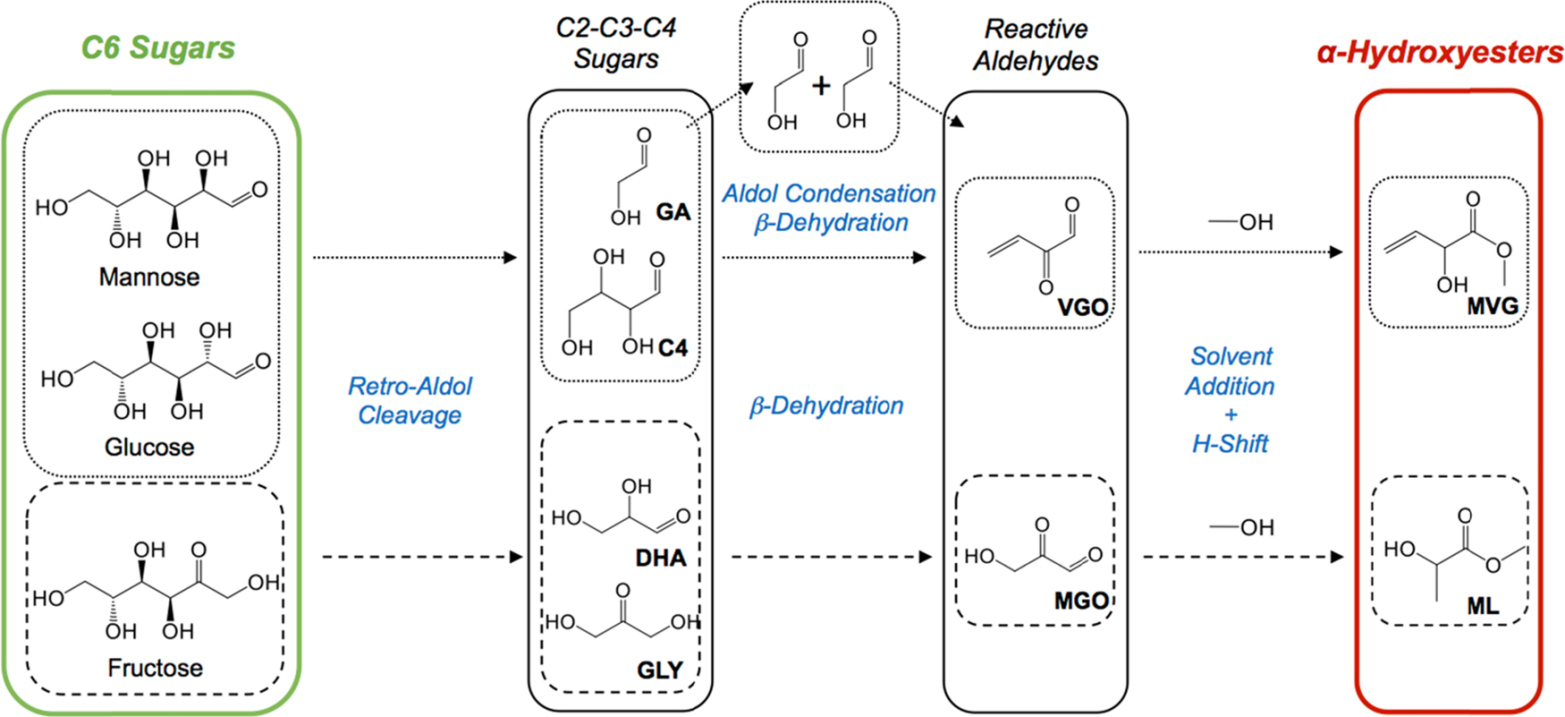
Reaction Pathway for the Retro-Aldol Cleavage of Glucose into α-Hydroxyesters (MVG and ML) Key: GLY = Glyceraldehyde, DHA = Dihydroxyacetone, MGO = Methylglyoxal, ML = Methyl lactate, C4 = Erythrose, GA = Glycolaldehyde, VGO = Vinylglyoxal, and MVG = Methyl Vinyl Glycolate

Considering the differing properties and performance among the Sn-Beta preparations, the potential of these catalysts for the continuous retro-aldol fragmentation of glucose to the α-hydroxyesters ML and MVG was evaluated. The different Sn-Beta preparations were first tested for the retro-aldol fragmentation of glucose in methanol at 160 °C, at a WHSV of 4.752 h^–1^. In this first instance, reactions were carried out without promoters to understand the intrinsic activity and stability of each catalyst, under conditions optimized by previous researchers.^15^ We note that a high WHSV was used to avoid operating in an excess catalyst regime, which can mask deactivation of the catalyst and hinder proper evaluation of the stability of the reaction system.^24,25^ However, the WHSV was deliberately set so as to attain a high degree of substrate conversion (>90% initial conversion), which is especially important toward attaining high yields of retro-aldol products, given the cascade nature of the reaction pathway (Scheme 2) and the conversion versus selectivity relationship of the reaction (vide infra).

**Figure 5:**
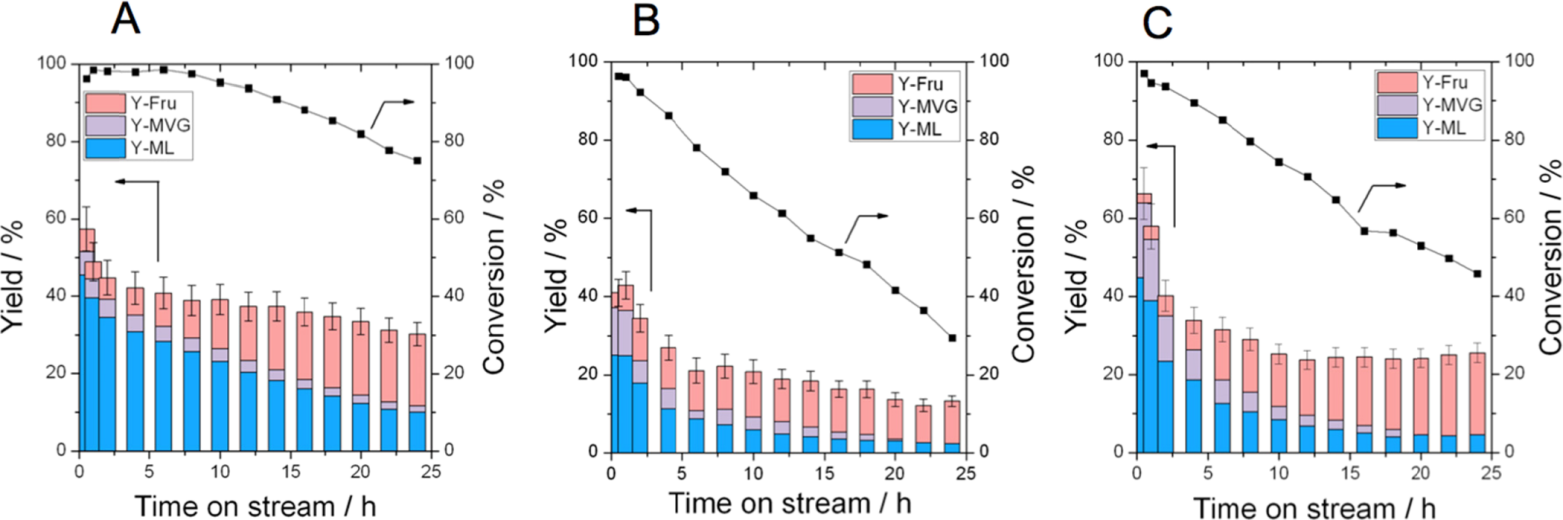
Time on stream data for glucose upgrading at 160 °C carried out in methanol by (A) Sn-Beta HDT, (B) Sn-Beta SSI, and (C) Sn-Beta PA. Conversion (black line) and yields of the major products (bar charts, including fructose, ML, and MVG) are reported as a function of time on stream. Reaction conditions: 1 wt % glucose in methanol, 1 mL min^–1^ flow rate, 100 mg of catalyst, 160 °C, resulting in a WHSV of 4.752 kg_glucose_ kg^–1^_catalyst_ h^–1^.

**Figure 6:**
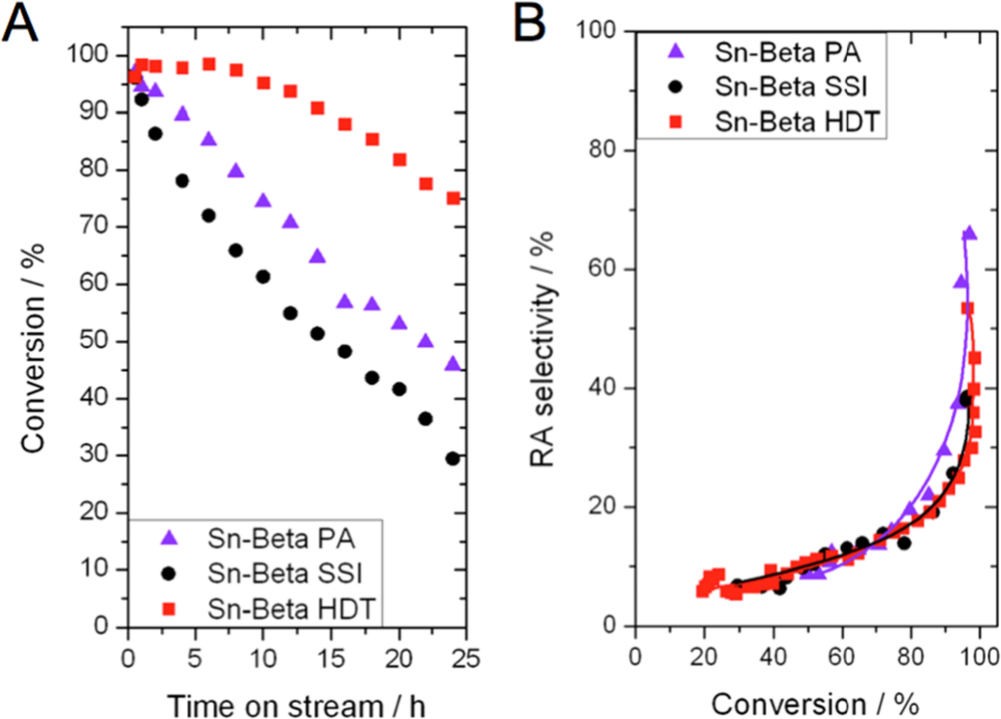
(A) Percentage of glucose converted as a function of time on stream for Sn-Beta HDT (red squares), Sn-Beta SSI (black circles), and Sn-Beta PA (violet triangles). (B) Conversion versus selectivity relationship for Sn-Beta HDT (red squares), Sn-Beta SSI (black circles), and Sn-Beta PA (violet triangles). Reaction conditions: 1 wt % glucose in methanol, 1 mL min^–1^ flow rate, 100 mg of catalyst, 160 °C, resulting in a WHSV of 4.752 kg_glucose_ kg^–1^_catalyst_ h^–1^.

The results of the kinetic experiments are shown in Figure 5 in which glucose conversion (eq 3) and individual product yields (eq 4) measured at the reactor outlet at that point of time are plotted as a function of the time on stream. The use of conversion and individual product yields was made to simplify interpretation of the data in the first instance, by allowing the overall carbon balance to be directly evident while also allowing the product distribution to be followed with time. This is especially helpful in the case of a complex cascade reaction, such as the retro-aldol fragmentation of glucose to α-hydroxyesters, where even a small decrease in substrate conversion markedly affects the calculated values of selectivity (eq 5). Nevertheless, conversion and selectivity dependencies are provided for further comparison in Figure 6.
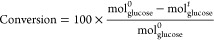
3
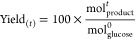
4
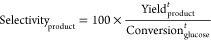
5

As can be seen (Figure 5), at 160 °C and a WHSV of 4.752 h^–1^, each Sn-Beta catalyst initially converted more than 90% of the substrate and produced significant yields of retro-aldol products (ML + MVG, henceforth referred to as RA products). Yet, clear differences in the overall performance of each catalyst were evident. Under these promoter-free conditions, Sn-Beta HDT was the most stable catalyst in terms of glucose conversion, followed by Sn-Beta PA and lastly Sn-Beta SSI (Figure 6A). In particular, Sn-Beta HDT lost around 25% of its activity over 24 h, whereas the postsynthetic materials experienced greater losses in performance (55 and 70% loss of activity for Sn-Beta PA and Sn-Beta SSI, respectively). This observation is in line with previous studies focused on glucose isomerization, which have indicated that hydrothermally prepared Sn-Beta possessed enhanced stability compared to Sn-Beta samples prepared by postsynthetic routes.^28^ This initial finding also confirms that preactivation of Sn-Beta improved the stability of the postsynthetic catalyst, though to a slightly lower extent than observed for glucose isomerization. Broadly speaking, all three catalysts exhibited comparable conversion versus selectivity relationships (Figure 6B), approaching 50% selectivity to RA products at full conversion. However, it is interesting to note how the initial yield of RA products was higher for Sn-Beta PA (65%) than for Sn-Beta HDT (52%), confirming the interesting potential of this catalyst for higher temperature operation (Figure 5). Interestingly, when Sn-Beta SSI was tested under the same conditions, the initial yield of RA products was only 37%, further demonstrating the superior performance of the preactivated material to that of unmodified Sn-Beta SSI in terms of activity and stability.

Despite the interesting yield of α-hydroxy esters achieved by these catalysts at initial time on stream, the steep deactivation exhibited by each sample represents a challenge for process intensification. One of the main causes for the deactivation of Sn-Beta in methanol is known to be the strong interaction between the Sn site of the catalyst and the reaction solvent.^16^ Recently, we have shown how the stability of Sn-Beta can be enhanced through the addition of small amounts of water to the reaction feed, since the presence of some water minimizes alkoxylation and/or condensation of the sites during continuous operation.^16,26^ However, the stabilizing role of water during retro-aldol fragmentation has only previously been observed for a postsynthetic Sn-Beta catalyst employing fructose as the substrate,^16^ and hence, its general applicability to stabilize other types of Sn-Beta catalysts (i.e., hydrothermal Sn-Beta catalysts) or retro-aldol reactions using glucose as the substrate has not yet been determined.

To understand the sensitivity of these catalysts to the addition of water as a stability promoter, the retro-aldol fragmentation of glucose was carried out in a solution of methanol/water (99:1 wt %, henceforth 99:1) (Figure 8). The principal effect observed upon adding a small amount of water to each reaction feed is that the stability of each catalyst is improved, as evidenced by the higher levels of conversion maintained by each catalyst during continuous operation over an initial 24 h period. Interestingly, although Sn-Beta HDT was still the most stable catalyst under these conditions, the stability of Sn-Beta PA and Sn-Beta SSI was enhanced to a much greater degree by the addition of water. Given that the beneficial effect of water primarily relates to the fact that small quantities of water limit the interaction of the active sites with the methanol solvent,^16,26^ this finding suggests that the major benefit of the hydrothermal preparation is that it results in the synthesis of a catalyst more resistant to deactivation by methanol in the first instance, which hence exhibits higher stability in the absence of additional water (Figure 6A). However, when small amounts of water are added to mitigate this primary deactivation mechanism, all Sn-Beta catalysts broadly exhibit similar levels of stability, resulting in all samples exhibiting broadly similar rates of deactivation when a methanol/water solution (99:1 v/v) was used as the solvent.

**Figure 7:**
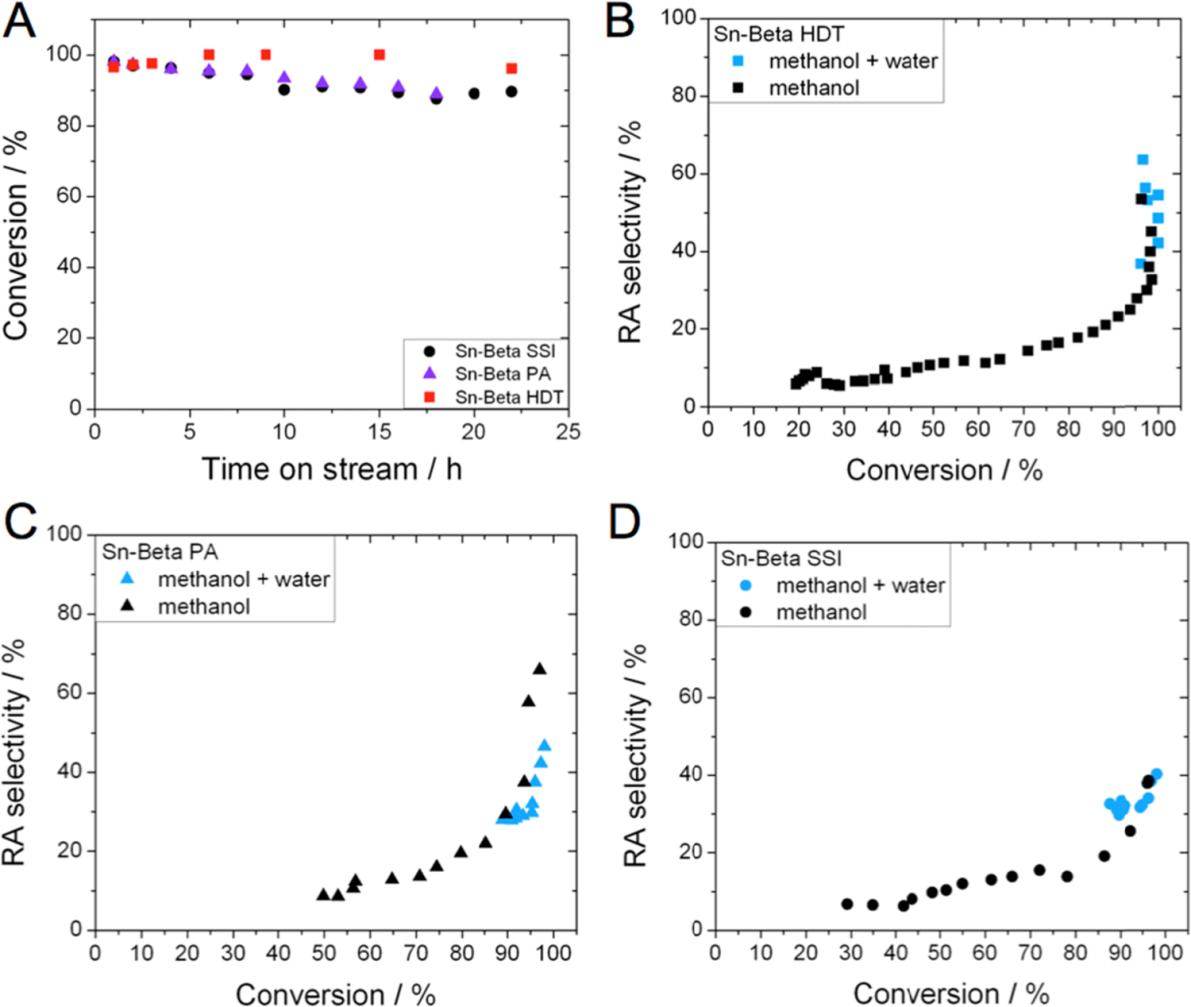
(A) Percentage of glucose converted as a function of time on stream during retro-aldol fragmentation in methanol/water, for Sn-Beta HDT (red squares), Sn-Beta SSI (black circles), and Sn-Beta PA (violet triangles). Conversion versus selectivity relationship for (B) Sn-Beta HDT, (C) Sn-Beta PA, and (D) Sn-Beta SSI in the absence (black) and presence (blue) of 1 wt % water in the reaction feed. Reaction conditions: 1 wt % glucose in methanol/water (99:1), 1 mL min^–1^ flow rate, 100 mg of catalyst, 160 °C, resulting in a WHSV of 4.752 kg_glucose_ kg^–1^_catalyst_ h^–1^.

Although all catalysts exhibited improved stability following the addition of 1 wt % water, the addition of water did not change the selectivity of the process toward the target retro-aldol products for any catalyst material. In particular, the conversion versus selectivity relationships were generally unchanged upon addition of 1 wt % water to the reaction feed for all catalysts (Figure 7B–D). As such, the maximum selectivity toward retro-aldol products was still higher for Sn-Beta HDT and Sn-Beta PA (ca. 50–60% at >90% substrate conversion) than for Sn-Beta SSI (<40% at >90% substrate conversion). We note that the low level of selectivity both reflects the cascade nature of the reaction process (Scheme 1) and further emphasizes the high reactivity and multifunctionality of Sn-Beta in being able to catalyze different reaction pathways.^12,30^ Consequently, despite the improved level of stability achieved upon addition of water, the selectivity of the process to retro-aldol products in methanol/water (99:1) still has margins for improvements.

Previous studies have shown how the addition of small quantities of alkali salts can improve the selectivity of certain Sn-Beta catalysts toward retro-aldol fragmentation.^12,21,22,30,55^ It has been proposed that the effect of alkali salts arises from their ability to exchange protons present in the active site environment, thereby minimizing the partial Brønsted acidity of Sn-Beta materials and favoring the retro-aldol fragmentation pathway.^21,55^ However, the susceptibility of different preparations of Sn-Beta catalysts for the alkali effect in continuous flow has not yet been evaluated. Thus, to determine the general susceptibility of these catalysts to alkali exchange, Sn-Beta HDT, Sn-Beta PA, and Sn-Beta SSI were tested for glucose upgrading at 160 °C in pure methanol and in the presence of alkali (KCl) but without added water. We note that KCl was used as the alkali promoter, as its beneficial concentration range is wider than that of other promoters with basic characters, such as K_2_CO_3_, and it can therefore be introduced into the feed in a slight excess without compromising performance, thus facilitating continuous operation.^22^

**Figure 8:**
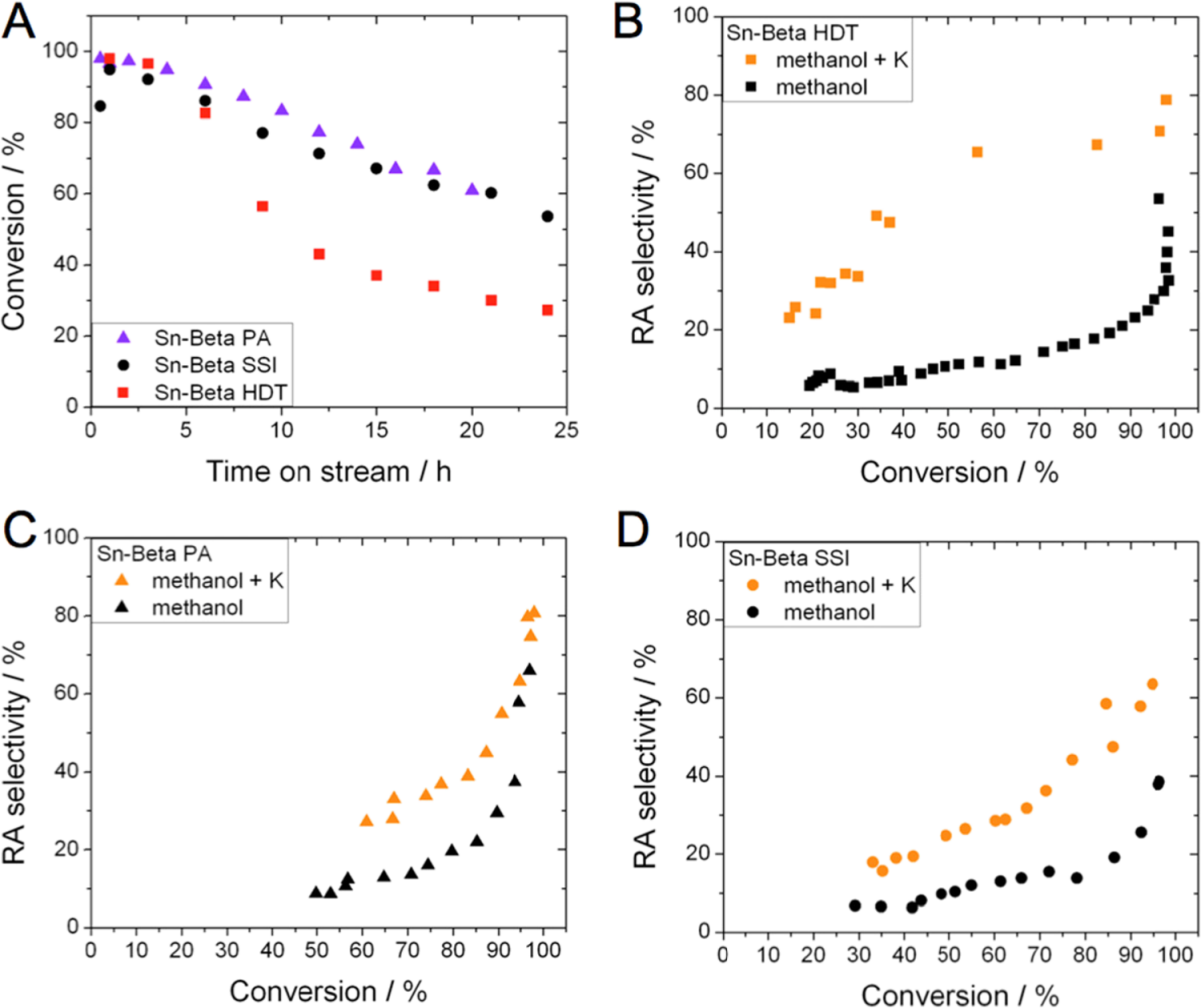
(A) Percentage of glucose converted as a function of time on stream during retro-aldol fragmentation of glucose in the presence of alkali, for Sn-Beta HDT (red squares), Sn-Beta SSI (black circles), and Sn-Beta PA (violet triangles). Conversion versus selectivity relationship for (B) Sn-Beta HDT, (C) Sn-Beta PA, and (D) Sn-Beta SSI in the absence (black) and presence (orange) of KCl in the reaction feed. Reaction conditions: 1 wt % glucose in methanol containing KCl (0.004 g L^–1^), 1 mL min^–1^ flow rate, 100 mg of catalyst, 160 °C, resulting in a WHSV of 4.752 kg_glucose_ kg^–1^_catalyst_ h^–1^.

**Figure 9:**
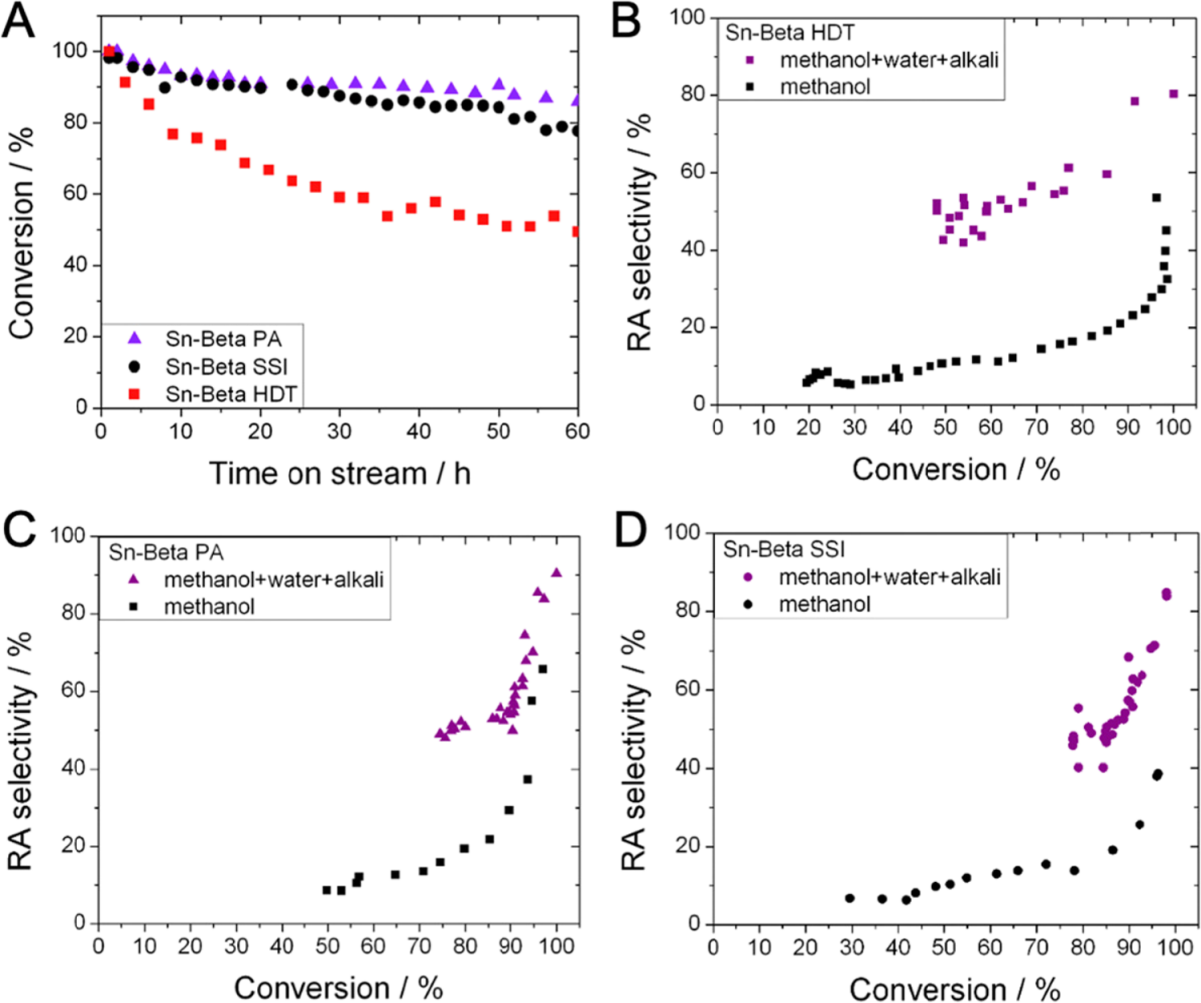
Percentage of glucose converted as a function of time on stream during retro-aldol fragmentation of glucose in methanol/water and in the presence of alkali, for Sn-Beta HDT (red squares), Sn-Beta SSI (black circles), and Sn-Beta PA (violet triangles). Conversion versus selectivity relationship for (B) Sn-Beta HDT, (C) Sn-Beta PA, and (D) Sn-Beta SSI in the absence (black) and presence (violet) of water and KCl in the reaction feed. Reaction conditions: 1 wt % glucose in methanol/water (99:1) containing KCl (0.004 g L^–1^), 1 mL min^–1^ flow rate, 100 mg of catalyst, 160 °C, resulting in a WHSV of 4.752 kg_glucose_ kg^–1^_catalyst_ h^–1^.

Figure 8A presents the conversion of glucose achieved over Sn-Beta HDT, Sn-Beta SSI and Sn-Beta PA as a function of time on stream, whereas Figure 8B–D presents the conversion versus selectivity dependence of each catalyst in the presence (orange symbols) and absence (black symbols) of alkali. Upon addition of alkali to the reaction feed, it is evident that the selectivity toward α-hydroxyester products increased dramatically for all catalyst materials (Figure 9B–D). Although the increase in selectivity toward retro-aldol products was the greatest for Sn-Beta HDT at lower levels of conversion, the highest overall RA selectivity was observed for Sn-Beta PA, which exhibited a RA selectivity greater than 80% at maximum conversion upon addition of KCl to the feed.

However, it is apparent that the addition of alkali salts to each reaction also leads to unexpected changes in the stability of each catalyst, when compared to the analogous reaction run in pure methanol without alkali salts present (comparing Figure 8A to Figure 6A). In particular, Sn-Beta SSI and Sn-Beta PA exhibited very slight improvements in stability upon addition of alkali. However, Sn-Beta HDT suffered a loss of stability upon inclusion of alkali and lost over 70% of its initial activity in 24 h, in contrast to less than 30% over the same time frame without addition of alkali. This has not previously been observed and is indicative of compatibility problems between the hydrothermal sample and alkali ions in the feed. Given the comparable active site properties of Sn-Beta PA and Sn-Beta HDT, the negative impact of alkali on the stability of Sn-Beta HDT is likely a consequence of the different hydrophilic and defect site density of Sn-Beta HDT relative to both postsynthetic catalysts. Since K^+^ has been shown to interact with both Sn sites and silanol groups, we hypothesize that the much lower defect site density of Sn-Beta HDT may indirectly result in over-titration of the Sn sites at the investigated K^+^/Sn ratios, since less K^+^ ions will be consumed via interaction with silanol groups.

The beneficial effect of alkali is thus clearly dependent on the precise choice of catalyst used for retro-aldol fragmentation. In particular, for both postsynthetic samples (Sn-Beta SSI and Sn-Beta PA), the addition of alkali was beneficial both to stability and selectivity, resulting in improved overall performance throughout the reaction period (Figure S2). In contrast, although the RA selectivity of Sn-Beta HDT was improved upon addition of alkali, its stability diminished substantially upon inclusion of alkali. As such, upon comparing the absolute RA product yield of Sn-Beta HDT in both the absence and presence of alkali, no clear benefit of adding alkali to the reaction after the first 8 h of the reaction can be seen, as the steeper rate of deactivation in the presence of alkali counteracts the improved selectivity (Figure S2).

To understand whether the stabilizing effect of water could be used in synergy with the effect of alkali, a final series of reactions was performed in methanol/water (99:1) in the presence of alkali salts (Figure 9). Although the copresence of water lowered the rates of deactivation for all catalysts, compared to the analogous experiments performed only with alkali (comparing Figures 9A to Figure 8A), the stability of Sn-Beta HDT was still negatively impacted by the presence of alkali (Figure 9A). Thus, under these conditions, Sn-Beta HDT displayed the lowest levels of stability, losing approximately 50% of its initial activity over the 60 h reaction period investigated (Figure 9A). In contrast, both postsynthetic samples retained over 80% of their initial performance over the same time period, with Sn-Beta PA retaining marginally more activity than Sn-Beta SSI.

The selectivity of each catalyst was also improved upon addition of alkali and water, but to a greater degree than observed with alkali alone. In fact, it is notable that the RA product selectivity plateaus at a much higher level for all catalysts, when alkali and water are employed in combination. Thus, maximum RA product selectivity greater than 90% was observed for both postsynthetic catalysts, which was slightly higher than recorded for Sn-Beta HDT, which reached a maximum selectivity of 80% at the highest levels of substrate conversion. In line with the data presented in Figure 8, the lower stability of Sn-Beta HDT in the presence of alkali counteracts its improved selectivity, resulting in there being no net-benefit regarding the addition of alkali to the feed for Sn-Beta HDT beyond the first 4–8 h under these particular conditions (Figure S3).

**Figure 10:**
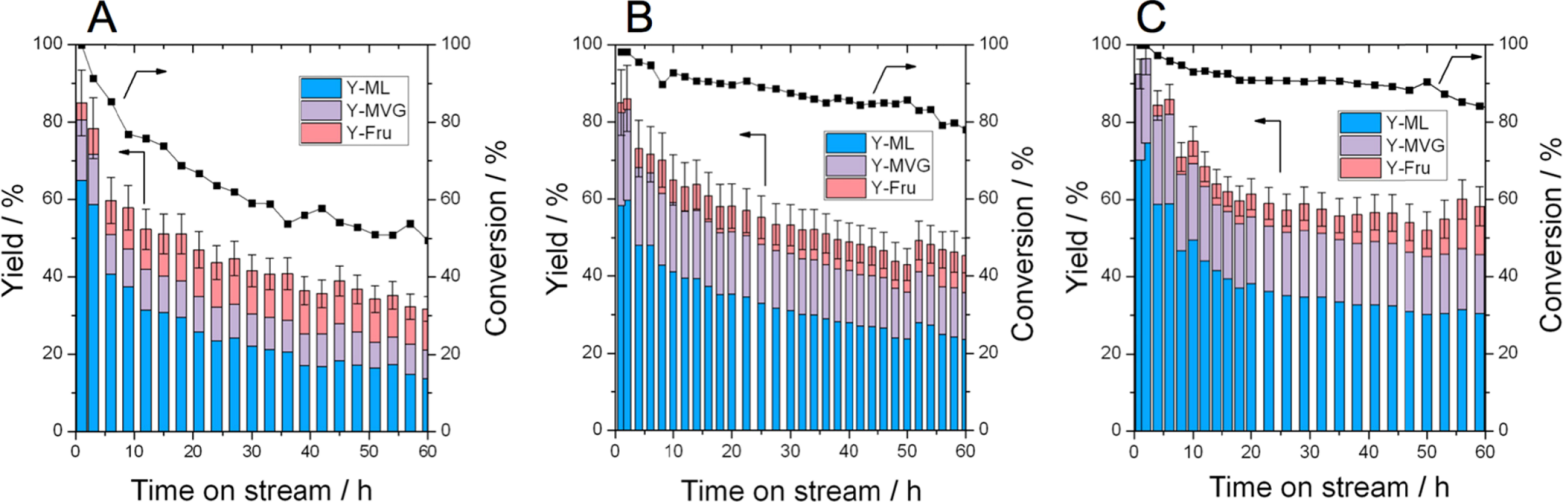
Time on stream data for glucose upgrading at 160 °C carried out in methanol/water (99:1) in the presence of alkali by (left) Sn-Beta HDT, (middle) Sn-Beta SSI, and (right) Sn-Beta PA, shown as conversion (black line) and yields (bar charts) as a function of time on stream. Reaction conditions: 1 wt % glucose in methanol/water (99:1), (KCl 0.004 g L^–1^), 1 mL min^–1^ flow rate, 100 mg of catalyst, 160 °C, WHSV: 4.752 kg_glucose_ kg^–1^_catalyst_ h^–1^.

As a consequence of the perturbations of water and alkali, all catalysts exhibit a large increase in overall yield to RA products, when compared to their analogous reactions performed without water and alkali (comparing Figure 10 to Figure 5). In particular, the initial yield of α-hydroxy esters products was approximately 80% for Sn-Beta HDT and Sn-Beta SSI and above 95% for Sn-Beta PA (75% yield for ML and >20% yield for MVG). As a consequence of their higher stability in the presence of alkali salts, the postsynthetic catalysts outperformed the hydrothermal catalyst by some margin, especially over longer operational periods. In particular, Sn-Beta PA was found to produce the highest yields of retro-aldol products throughout the reaction period, which reached a plateau of approximately 45–50% after the first 24 h on stream (Figure 10).

**Figure 11:**
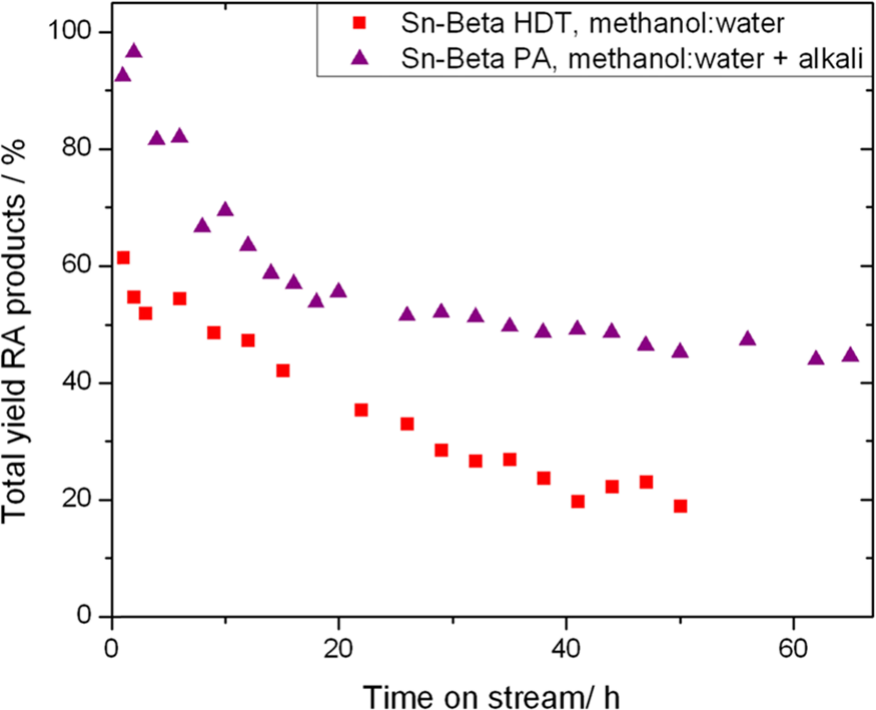
Yield of retro-aldol (RA) products (ML + MVG) as a function of time on stream during retro-aldol fragmentation of glucose using Sn-Beta HDT (methanol/water, no alkali) and Sn-Beta PA (methanol/water and alkali). Reaction conditions: 1 wt % glucose in methanol/water (99:1), 1 mL min^–1^ flow rate, 100 mg of catalyst, 160 °C. Where applicable, i.e., for Sn-Beta PA, KCl was present at 0.004 g L^–1^.

When determining the optimal catalyst for a process, it is important to consider activity, selectivity, and stability, all of which are dependent on the precise properties of a catalyst and the reaction conditions used.^23−25^ For Sn-Beta HDT, better performance is achieved when retro-aldol fragmentation is performed in methanol/water but without addition of alkali to the feed, given that alkali negatively affects the stability of the hydrothermal catalyst. In contrast, for both postsynthetic samples, the addition of alkali is beneficial to both stability and selectivity, and hence, a better performance is achieved in methanol/water with alkali present. However, between these two general selections, the latter option results in much higher RA product yields being achieved, particularly over extended operational periods. This is exemplified in Figure 11, which compares the combined RA product yield produced using the best-performing postsynthetic catalyst (Sn-Beta PA) in the presence of water and alkali, to the combined RA yield achieved using Sn-Beta HDT in the presence of the water additive alone, under otherwise identical reaction conditions.

Although operating at submaximal conversion is essential toward obtaining accurate insight into the stability of a catalyst, in the case of retro-aldol fragmentation, this approach clearly hinders the achievement of high yields, given the steep conversion versus selectivity relationship of the reaction. Even so, the maximum combined RA yields achieved by each of the catalysts described above are far above those previously reported for this reaction when using glucose as the substrate, which have typically been in the region of 45–60% (Table S1). Although most previous studies report only ML yields and do not explicitly report on the production of MVG, the ML yields achieved over Sn-Beta PA are still higher than previously reported and thus still represent a new benchmark in terms of retro-aldol fragmentation reactions using glucose as the substrate.

In addition to providing important opportunities to probe the stability of heterogeneous catalysts, continuous PFRs offer several other advantages over batch reactors, including improved mass and heat transfer, increased safety, better scalability, and smaller reactor volumes. As a consequence of these benefits, employing these catalysts in such an operational mode (PFR operating at high WHSV (4.752 h^–1^, vide supra)) results in substantial increases in space–time-yields. In particular, the values of space–time-yield achieved by use of Sn-Beta PA under its most suitable conditions (methanol/water solvent with the alkali additive) are over 2 orders of magnitudes higher than previously reported for this particular reaction, where most studies have been carried out in batch reactors (Table S1).^12,16,19,27,37,57,58^ Alongside the increased overall yields of retro-aldol products (>95%, registered at full conversion of glucose), the performances of the catalysts explored in this study are thus substantially higher than the best results reported to date, demonstrating the importance of optimizing the global reaction system (i.e., catalyst and conditions) in unison.

## Conclusions

In this manuscript, postsynthetic Sn-Beta catalysts were compared in performance to hydrothermally prepared Sn-Beta for the isomerization of glucose to fructose at 110 °C and more thoroughly for the retro-aldol fragmentation of glucose to α-hydroxyesters at 160 °C. Initially, the best levels of performance were observed for the Sn-Beta catalyst prepared by hydrothermal synthesis, in line with the majority of previous research in the open literature. However, more thorough evaluation of the performances of these catalyst, in the continuous regime and in the presence of activity promoters (water and alkali), led to the observation that postsynthetic Sn-Beta samples exhibit the best levels of performance, when activity, selectivity, and stability are fully considered. In particular, the highest levels of retro-aldol product yield and stability are obtained with postsynthetic Sn-Beta catalysts preactivated in a flow of methanol prior to reaction. The active sites of this catalyst resemble those found in hydrothermal Sn-Beta, but its hydrophilicity and defect site density are more comparable to other postsynthetic analogues. Until now, the best performance for the conversion of glucose was achieved by the hydrothermal preparations of Sn-Beta, so the preactivation protocol represents an innovative and simple post synthetic methodology to access new Sn-Beta materials with an improved performance while providing important guidelines with respect to tailoring future Sn-Beta catalysts. More broadly, this study implies that the performances of different Sn-Beta catalysts intimately depend on the precise operational conditions employed, since different catalysts respond to different conditions and promoters to different degrees and in a very diverse manner. In fact, the responses of the catalysts to promoters appear to not only depend upon the nature of the active sites but also the active site environment and the properties of the zeolite lattice, making these relationships nonpredictable. Thus, along with demonstrating that postsynthetic Sn-Beta catalysts are more favorable for α-hydroxyester formation than hydrothermal analogues, this suggests that future development of Sn-Beta materials should not solely focus on pure activity and selectivity measurements under idealized conditions but should also study the susceptibility of each catalyst to the process promoters (alkali salts and water) under more realistic operational conditions. Thanks to this approach, we achieve α-hydroxyester yields over 90% when employing postsynthetic Sn-Beta catalysts that are preactivated in a flow of methanol prior to reaction. We also demonstrate continuous operation of the catalyst for over 60 h, providing important information regarding the stability of these catalysts under continuous processing conditions. In doing so, we obtained space–time-yields over two orders of magnitude higher than previously reported for this reaction while achieving unmatched yields of retro-aldol products at early stages of the reaction.

## Experimental Details

### Catalyst Synthesis

Hydrothermal synthesis of Sn-Beta was performed following a procedure described in the literature.^42^ First, 30.6 g of tetraethyl orthosilicate (Sigma Aldrich, 98%) was added to 33.1 g of tetraethylammonium hydroxide (Sigma Aldrich, 35%) under careful stirring, forming a two-phase system. After 60–90 min, one phase was obtained and the desired amount of the metal source (SnCl_4_·5H_2_O, Sigma Aldrich >99.5%) dissolved in 2.0 mL of H_2_O was added dropwise. The solution was then left for several hours under stirring until a viscous gel was formed. The gel was finalized by the addition of 3.1 g of hydrofluoric acid (Fischer Chemicals 50%) in 1.6 g of demineralized H_2_O, yielding a solid gel with the molar composition: 1.0Si, 1:0.005Sn, 1:0.02Cl^–^, 1:0.55TEA^+^, 1:0.55F^–^, and 1:7.5H_2_O. The obtained gel was transferred in a Teflon-lined stainless steel autoclave and kept for 7 days at 140 °C to crystallize. The obtained crystals were filtered and washed with deionized water. Calcination at 550 °C (2 °C min^– 1^) for 6 h under static air was carried out to remove the organic template.

Postsynthetic synthesis of Sn-Beta (Sn-Beta SSI) was carried out on the commercial zeolite Al-Βeta (Zeolyst, NH_4_^+^-form, Si/Al = 19). This was dealuminated by treatment in HNO_3_ solution (13 M HNO_3_, (VWR, Technical Chemicals, 68%) 100 °C, 20 mL g^–1^ zeolite, 20 h). Solid-state incorporation was achieved by grinding an appropriate amount of tin(II) acetate with the necessary amount of dealuminated zeolite for 10 min in a pestle and mortar. Following this procedure, the sample was heated in a combustion furnace (Carbolite MTF12/38/400) to 550 °C (10 °C min^–1^ ramp rate) first in a flow of N_2_ (3 h) and subsequently air (3 h) for a total of 6 h. Gas flow rates of 60 mL min^–1^ were employed at each stage.

Sn-Beta PA was obtained by treating Sn-Beta SSI at 110 °C in a continuous flow of methanol (VWR, methanol, >99.9 wt % purity) in a continuous reactor, at a flow rate of 1.5 mL min^–1^ for 0.5 h. The methanol-treated catalyst was immediately calcined in air at 550 °C for 3 h (10 °C min^–1^) following treatment in the flow reactor.

### Kinetic Studies

Continuous glucose upgrading reactions were performed in a plug flow, stainless steel, tubular reactor. The catalyst was pelletized (size fraction 63 and 77 μm) and densely packed into a 1/4 inch stainless steel tube (4.1 mm internal diameter). Two plugs of quartz wool and a frit of 0.5 μm held the catalyst in location. Temperature control was achieved with a thermostatted oil bath at the desired reaction temperature, and pressurization was achieved by means of a backpressure regulator. Continuous glucose isomerization experiments were performed at 110 °C, using a reaction feed of 1 wt % glucose in methanol, flow rates of 1.5 mL min^–1^ (Sn-Beta HDT), 1.0 mL min^–1^ (Sn-Beta PA), and 0.6 mL min^–1^ (Sn-Beta SSI), and 100 mg of catalyst in each case. Retro-aldol fragmentation reactions were performed at 160 °C, using a reaction feed of 1 wt % glucose in solvent (methanol or methanol/water (99:1)), at flow rates of 1 mL min^–1^. The same catalyst mass (100 mg) was used in every retro-aldol fragmentation reaction. Aliquots of the reaction solutions were taken periodically from a sampling valve placed after the reactor and were analyzed with an Agilent 1260 Infinity HPLC equipped with a Hi-Plex Ca column and ELS detector and quantified against an external standard (sorbitol) added to the sample prior the injection. Samples were also measured by GC-FID (Agilent 8890) for quantification of the C3 and C4 products.

### Catalyst Characterization

XRD spectra were acquired using a PANalytical X’PertPRO X-ray diffractometer, using a CuKα radiation source (40 kV and 30 mA). Diffraction patterns were recorded between 6 and 55° 2θ (step size 0.0167°, time/step = 150 s, and total time = 1 h). Specific surface area was determined from nitrogen adsorption using the BET equation, and microporous volume was determined from nitrogen adsorption isotherms using the t-plot method. Porosimetry measurements were performed with a Quantachrome Autosorb-iQ-MP/XR, and samples were degassed prior to use (115 °C, 6 h, N_2_ flow). Vapor adsorption experiments were performed on the same instrument maintaining the sample at 20° and adsorbing water from 0 to 0.7 *P*/*P*_0_. All the samples were nonenriched and were measured with a Bruker Avance III HD spectrometer at operating frequencies of 400 and 149 MHz for ^1^H, and ^119^Sn, respectively. Typically, between 50 and 100 mg of solid sample was packed in a 4 mm rotor, which was spun at ±12,000 Hz. Other spinning frequencies were employed for identification of spinning side bands. For ^119^Sn MAS NMR, samples were measured by the CMPG method as described in ref (56). Spectra were acquired both in direct excitation and cross polarization modes. A recycle delay time of 135 s was applied, as this provides a (semi)quantitative representation of the various Sn species present in each catalyst. DRIFT spectroscopy analyses were performed in a Harrick praying mantis cell. The spectra were recorded on a Bruker Tensor Spectrometer over a range of 4000–650 cm^–1^ at a resolution of 2 cm^–1^.

## Supporting Information Available

Supporting kinetic data, characterization information, and literature comparisons (PDF)
