# Supplementary material for: High-Productivity Continuous Conversion of Glucose to α-Hydroxy Esters over Postsynthetic and Hydrothermal Sn-Beta Catalysts
Source: ACS Sustain Chem Eng. 2022 Mar 30;10(14):4391–403. doi: 10.1021/acssuschemeng.1c06989 (PMC9007564; doi:10.1021/acssuschemeng.1c06989)
Supplement: Supplementary file 1 — sc1c06989_si_001.pdf [file sc1c06989_si_001.pdf]

Supporting information for:

High-productivity continuous conversion of glucose to α-hydroxy esters over post-synthetic and hydrothermal Sn-Beta catalysts

Luca Botti,[1] Ricardo Navar,[2] Søren Tolborg,[3] Juan S. Martínez-Espín[3] and Ceri Hammond[1]*

[1]Department of Chemical Engineering, Imperial College London, London, SW7 2AZ, UK

[2]Cardiff Catalysis Institute, Cardiff University, Park Place, Cardiff, CF10 3AT, UK

[3]Biobased Chemicals R&D, Haldor Topsøe A/S, Haldor Topsøes Allé 1, 2800-Kgs. Lyngby, Denmark

E-mail: ceri.hammond@imperial.ac.uk

Keywords Biomass · Zeolite · Continuous · Retro-aldol · Sn-Beta

5 pages in total, including 3 figures and 1 table, and additional references.

Table of contents.

Page S1: Cover page and Table of Contents

Page S2: Figure S1: X-ray Diffraction Analysis of various Sn-Beta catalysts.

Page S2: Figure S2: Additional kinetic data for various Sn-Beta catalysts.

Page S3: Figure S3: Additional kinetic data for various Sn-Beta catalysts.

Page S4: Table S1: Comparison of kinetic performances of this work to those in the open literature.

Page S4-5: References for supporting information.

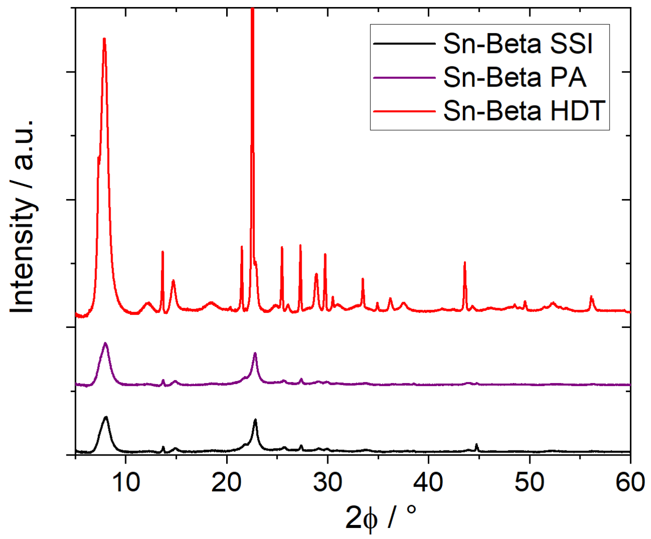

Figure S1. X-Ray Diffraction analysis for Sn-Beta HDT (top, red line), Sn-Beta PA (middle, purple line) and Sn-Beta SSI (bottom, black line).

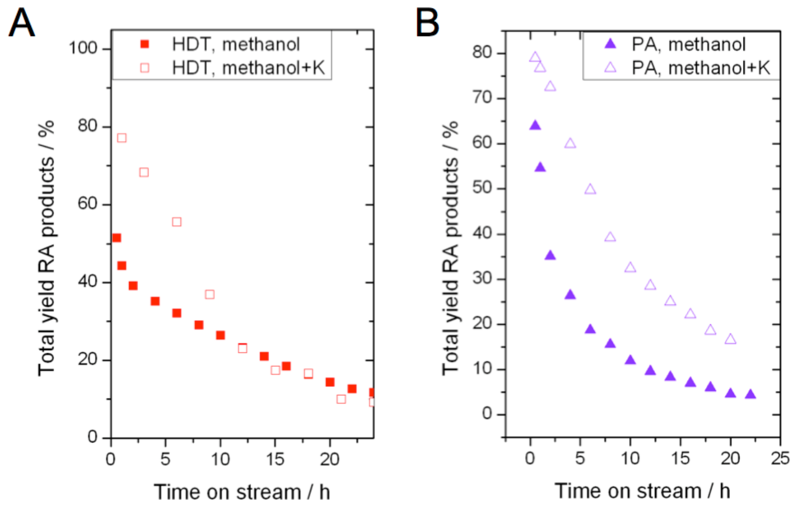

Figure S2. Yield of retro-aldol (RA) products (MVG+ML) generated by glucose cleavage at 160°C in pure methanol in the absence of (solid symbol) and in presence of alkali salts (empty symbols) for **(A)** Sn-Beta HDT and **(B)** Sn-Beta PA. Reaction conditions: 1 wt. % glucose in methanol, 1 mL min^{-1} flow rate, 100 mg catalyst, 160°C, WHSV of 4.752 $kg_{glucose}$ $kg^{-1}_{catalyst}$ h^{-1}. Where applicable, KCl was added to the feed directly at 0.004 g L^{-1}.

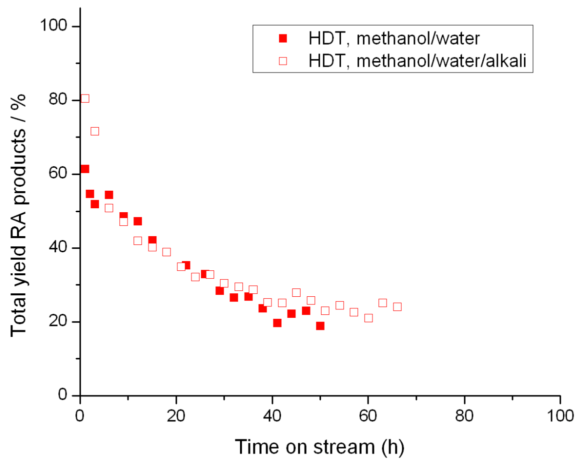

Figure S3. Yield of retro-aldol (RA) products (MVG+ML) generated by glucose cleavage at 160°C in methanol and water (solid symbol) and in methanol, water and alkali salts (empty symbols). Reaction conditions: 1 wt. % glucose in methanol and containing KCl (0.004 g L^{-1}), and 1 wt. % glucose in methanol:water (99:1), 1 mL min-1 flow rate, 100 mg catalyst, 160°C, WHSV of 4.752 $kg_{glucose}$ $kg^{-1}_{catalyst}$ h^{-1}. Where applicable, KCl was added to the feed directly at 0.004 g L^{-1}.

Table S1. Comparison of performance achieved in this work and those reported in previous studies.

Catalyst	Reactor configuration	Substrate	Maximum yield ML (%)	Maximum yield MVG (%)	Space-time-yield @ maximum yield ($g\ cm^{-3}\ h^{-1}$)	Reference
Sn-Beta PA	C	G	75	>20	2.7	This work
Sn-Beta HDT	C	G	45	N/A	5.5×10^{-2}	1
Sn-Beta SSI	C	F	60	20	3.6×10^{-1}	2
Sn-MWW	B	G	50	N/A	2.9×10^{-4}	3
Hierarchical Sn-Beta	B	G	50	N/A	1.2×10^{-3}	4
Hierarchical Sn-Beta	B	G	52	N/A	4.2×10^{-4}	5
Sn(OTf)	B	G	28	12	8.9×10^{-4}	6
Sn-Beta HDT Alkali	B	G	50	15	2.28×10^{-2}	7

[a]C = continuous flow. B = batch. G = glucose. F = fructose.

N/A = Not Applicable, value not reported

References for Supporting Information

(1) Zhang, Y.; Luo, H.; Zhao, X.; Zhu, L.; Miao, G.; Wang, H.; Li, S.; Kong, L. Continuous Conversion of Glucose into Methyl Lactate over the Sn-Beta Zeolite: Catalytic Performance and Activity Insight. *Ind. Eng. Chem. Res.* **2020**, *59* (39), 17365–17372.

(2) Padovan, D.; Tolborg, S.; Botti, L.; Taarning, E.; Sádaba, I.; Hammond, C. Overcoming Catalyst Deactivation during the Continuous Conversion of Sugars to Chemicals: Maximising the Performance of Sn-Beta with a Little Drop of Water. *React. Chem. Eng.* **2018**, *3*, 155–163.

(3) Guo, Q.; Fan, F.; Pidko, E. A.; Van Der Graaff, W. N. P.; Feng, Z.; Li, C.; Hensen, E. J. M. Highly Active and Recyclable Sn-MWW Zeolite Catalyst for Sugar Conversion to Methyl Lactate and Lactic Acid. *ChemSusChem* **2013**, *6*, 1352–1356.

(4) Tang, B.; Li, S.; Song, W. C.; Yang, E. C.; Zhao, X. J.; Guan, N.; Li, L. Fabrication of Hierarchical Sn-Beta Zeolite as Efficient Catalyst for Conversion of Cellulosic Sugar to Methyl Lactate. *ACS Sustain. Chem. Eng.* **2020**, *8* (9), 3796–3808.

(5) Zhang, J.; Wang, L.; Wang, G.; Chen, F.; Zhu, J.; Wang, C.; Bian, C.; Pan, S.; Xiao, F. S. Hierarchical Sn-Beta Zeolite Catalyst for the Conversion of Sugars to Alkyl Lactates. *ACS Sustain. Chem. Eng.* **2017**, *5*, 3123–3131

(6) Dusselier, M.; De Clercq, R.; Cornelis, R.; Sels, B. F. Tin Triflate-Catalyzed Conversion of Cellulose to Valuable (α-Hydroxy-) Esters. *Catal. Today* **2017**, *279*, 339–344.

(7) Tolborg, S.; Meier, S.; Sádaba, I.; Elliot, S. G.; Kristensen, S. K.; Saravanamurugan, S.; Riisager, A.; Fristrup, P.; Skrydstrup, T.; Taarning, E. Tin-Containing Silicates: Identification of a Glycolytic Pathway via 3-Deoxyglucosone. *Green Chem.* **2016**, *18*, 3360–3369.
